# Compliance With the Use of Low-Vision Aids in a Greek Population: An Explorative Study

**DOI:** 10.7759/cureus.42730

**Published:** 2023-07-31

**Authors:** Konstantinos Oikonomidis, Stavroula Almpanidou, Persefoni Talimtzi, Angeliki Kakavouti-Doudou, Spyridon M Metaxas, Vasileios Karampatakis

**Affiliations:** 1 Laboratory of Experimental Ophthalmology, School of Medicine, Aristotle University of Thessaloniki, Thessaloniki, GRC; 2 1st Department of Ophthalmology, School of Medicine, Aristotle University of Thessaloniki, Thessaloniki, GRC; 3 2nd Department of Otorhinolaryngology, School of Medicine, Aristotle University of Thessaloniki, Thessaloniki, GRC; 4 Laboratory of Experimental Ophthalmology, Aristotle University of Thessaloniki, Thessaloniki, GRC

**Keywords:** training, quality of life, low vision aids, rehabilitation, low vision

## Abstract

The aim of this study is to investigate the compliance with low-vision aids (LVAs) among patients with low vision (LV) in a Greek population. An explorative study was conducted in a sample of patients with LV attending our outpatient unit at the School of Medicine, Aristotle University of Thessaloniki, Thessaloniki, Greece. Patients’ demographics and daily visual demands were recorded, and they were administered with the National Eye Institute Visual Function Questionnaire-25 (VFQ-25) at baseline. Participants were trained in the use of a wide range of LVAs before their prescription. Evaluation of the use of the LVAs was conducted at one year after the baseline using a structured phone survey. A total of 100 LV patients were included, with 68% of them being older than 65 years and 50 being males. The main cause of LV (57.0%) was age-related macular degeneration, and the mean VFQ-25 score at baseline was 49.2 (SD= 17.8). Overall, 75 patients had been prescribed LVAs, with 76.0% of these patients preferring an optical aid. The vast majority (98.7%) of these patients stated using the LVA one year after the baseline, and 62.1% of them reported using the aid often to very often. Significantly, 76% of these patients reported that their quality of life was positively affected by the use of the aid, and 97.3% would recommend the use of LVA to another individual with the same problem. Providing appropriate training before the prescription is of high significance to improve the rate of compliance with the use of LVAs. These results can be used to develop appropriate strategies in this field.

## Introduction

Low vision (LV) refers to patients who are not legally blind but experience moderate-to-severe vision loss [1,2]. In 2017, the World Health Organization (WHO) estimated that 253 million people worldwide were living with visual impairment, from which 36 million were legally blind, numbers that have already increased to 285 million and 39 million, respectively [1,2]. Taking into account that the population is constantly aging, shortly the percentage of people with LV is going to increase. LV was defined as “visual acuity of less than 6/18 but equal to or better than 3/60, or a corresponding visual field loss to less than 20°, in the better eye with the best possible correction” during the 10th revision of the WHO International Statistical Classification of Diseases, Injuries, and Causes of Death [3]. Thus, an individual with LV has impaired vision that cannot be corrected with therapeutic interventions [3].

In developed countries, LV is most commonly caused by age-related macular degeneration (AMD), glaucoma, and diabetic retinopathy (DR) [4], causing devastating consequences in all aspects of daily life. Patients with LV face difficulties with activities of daily living, which leads to loss of their independence and consequently decreases their quality of life (QoL) [5,6]. Until today, there is no curative therapeutic intervention available for most of the diseases causing LV [7]. In other words, the use of low-vision aids (LVAs) and participation in adequate rehabilitation programs are fundamental for visually impaired individuals to cope with their handicaps.

Rehabilitation programs aim to train patients with LV in the proper use of LVAs to exploit their residual vision and regain some degree of their functionality and independence. According to previous studies, LVAs constitute a fundamental form of intervention in LV rehabilitation programs as they substantially support daily activities such as reading, watching TV, money recognizing, etc. [4] Magnification devices such as hand, stand, electronic magnifiers, and telescopes are among the most common used LVAs [4,8].

Although assistive devices might be considered a highly valuable intervention toward LV rehabilitation, facilitating the management of daily disability, the compliance with the use of such devices may be challenging [9]. Despite the broad availability of LVAs such as magnifiers and electronic reading devices, many studies support that they are underutilized and that patients with LV have little awareness of these devices [10,11]. In a recent study by Gothwal and Sharma in adults with LV, patients experiencing no change in their QoL were more prone to abandon the prescribed LV device than those who reported some change resulting from the use of the device. The complexity and the size of such devices along with other psychological factors were the most frequently reported reasons for non-compliance with the use of LVAs [12].

Toward developing strategies to increase compliance with the use of LVAs, experts in the field proposed that LVAs should be task-specific and prescribed according to the needs of LV patients. Furthermore, a training program in the appropriate use of LVAs should be provided by the rehabilitation services before their prescription [13-18]. The study aimed to investigate compliance with the use of LVAs in a Greek cohort of LV patients attending a training protocol before the prescription of LVAs. Parameters such as QoL, visual acuity, educational status, type of disease, and age, which may affect compliance with these aids at baseline and before prescription, were also investigated.

## Materials and methods

Participants

An explorative study was conducted at the Laboratory of Experimental Ophthalmology at the School of Medicine, Aristotle University of Thessaloniki, Thessaloniki, Greece. Patients with LV were recruited from our outpatient unit for patients with visual impairment (convenience sample). Participants were adults (age 18 years and over) with LV according to the definition of LV by WHO [3]. This research was conducted in compliance with the Declaration of Helsinki, and all study procedures were approved by the Committee for Bioethics and Ethics, Medical Department, Aristotle University of Thessaloniki (code 4.638/21.02.2022). Informed written consent was obtained from participants after an explanation of the nature of the study. Participants who were unable to provide informed consent and those who suffered from severe motor and other severe systemic comorbidities (end-stage cancer, end-stage liver disease, etc.) were excluded from the study.

Procedure

Briefing sessions regarding the appropriate use of LVAs were organized and conducted. LVAs included optical devices such as magnifiers (illuminated and nonilluminated) and electronic devices such as portable and desktop video magnifiers. During the briefing sessions, participants were asked to report daily activities that were substantially influenced by their visual impairment to define the frame of assistance that could be achieved with the use of the available LVAs (Tables 1, 2). Special attention was given to the training of patients in the use of the LVAs, and the primary goal of the whole process was to determine the patients’ perspectives regarding the range of activities in which the LVAs could be comfortably used. Specifically, patients were instructed on how to handle the LVA, and they had to use it at least for 1 hour following the instructions of the ophthalmologist. The training protocol was based on a previously published protocol regarding the training of LV patients in the proper use of closed-circuit televisions (CCTV) [19] and according to the Low Vision Intervention Trial (LOVIT) [20] (Table 1).

**Table 1:** Standard training protocol in the use of LVAs during the sessions LVA, low-vision aid

Available LVAs	Handheld magnifier with and without illumination (magnification range from x3 to x10), electronic optical reader (magnification range from x3 to x10), laptop electronic magnifiers, head-mounted distance glasses (x2.1 magnification), high-plus reading lenses
Basic operation instructions	Explanation of relative advantages and limitations of each aid, definition of the activities each aid could be used for, on/off switch, magnification selection, position of lights, contrast selection
Ergonomics	Posture for using/working with the LVAs
Reading	Basic reading assignments (e.g., reading numbers/words/small sentences); reading bills, books, magazines, medicine bottles, etc.
Watching pictures/TV	Watching pictures and photographs, watching TV at different distances
Other interests and hobbies of the patients	Pets, sports, painting, etc.
Number and duration of sessions	Adjusted by the examiner according to the patients’ needs
Format of sessions	Face-to-face evaluation, counseling, and training

**Table 2:** Self-reported daily activities that were affected by the LV participants (N=100) LV, low vision

Daily activities	Ν (%)
Technology-communication	32 (32.0)
Cooking	43 (43.0)
Watching television	44 (44.0)
Self-care	27 (27.0)
Social life	38 (38.0)
Housekeeping	39 (39.0)
Reading	56 (56.0)
Going out	39 (39.0)
Travelling	12 (12.0)
Shopping	22 (22.0)
Gardening	12 (12.0)
Work/education	28 (28.0)
Pets care	4 (4.0)
Driving	6 (6.0)

Tools and methods

Participants' best-corrected visual acuity (BCVA) was evaluated using the Early Treatment Diabetic Retinopathy Study (ETDRS), and the results were converted to the logarithm of the minimum angle of resolution (logMAR) [21]. Visual acuity of the better-seeing eye was used for the analysis. The variables examined during the baseline session (time point 1) regarding the decision of participants to receive an LVA were age, educational level, gender, visual acuity (BCVA), the underlying cause of LV, and the QoL, as assessed at baseline (time point 1) using the Greek version of the Visual Function Questionnaire-25 (VFQ-25). [22,23] The VFQ-25 questionnaire consists of 25 items and is one of the most frequently used questionnaires to assess vision-related QoL (VRQoL). The scoring method proposed by developers includes 12 subscores and one average VRQoL score, ranging from 0 = worst to 100 = best [22,23].

At the end of the final individualized session, the preferred LVA(s) was/were prescribed, and large-print instructions were provided including the activities in which each aid could be used, the care of the aid, the type of lighting conditions, and instructions to wear their reading glasses when using the aid [24] The use of the prescribed LVA(s) was evaluated in one-year interval (time point 2) using a structured phone survey based on a previously reported research by Demirkilinc et al. [25]. The questionnaire evaluates the compliance with the LVA(s) use and was administered by phone. The question that defined the use of LVA(s) was asking participants: “Have you used the device?” and the actual use was defined as a binary variable (yes/no question). Abandonment of the prescribed device was defined as non-use in the past three months [26]. Other quantitative questions used referred to the frequency of use (very often/often/sometimes/rarely/never) and the self-reported change of QoL: “How was your quality of life affected” (positively/slightly positively/no difference/slightly negatively/negatively). Other questions referred to the self-reported benefit from the use of the aid: “Do you feel that you benefit from the aid?” and the answer was defined as a binary variable (yes/no question) and if they would recommend the aid to other people with the same problem the answer was defined as a binary variable (yes/no question). The feedback questionnaire is presented in the Appendix [25].

Statistical analysis

Descriptive statistics were calculated using the mean and standard deviation or median and interquartile range for continuous variables, while frequencies (N) and percentages (%) were used for categorical variables. Data normality was tested using the Shapiro-Wilk test. The chi-square test or Fisher’s exact test was used for correlation of the compliance with the LVA (no, yes) at baseline with age (<45 years, 45-65 years, >65 years), gender (male, female), education (mandatory, high school, university), diagnosis (AMD, glaucoma, DR, retinitis pigmentosa, other), and BCVA (logMAR units). The independent samples t-test or the Mann-Whitney U test was used for the correlation between the compliance with the LVA at baseline and the different scores of the VFQ-25 questionnaire. Statistical analysis was performed using SPSS Statistics Version 27 (IBM Corp., Armonk, NY). The statistical significance level was set at p<0.05.

## Results

**Figure 1:**
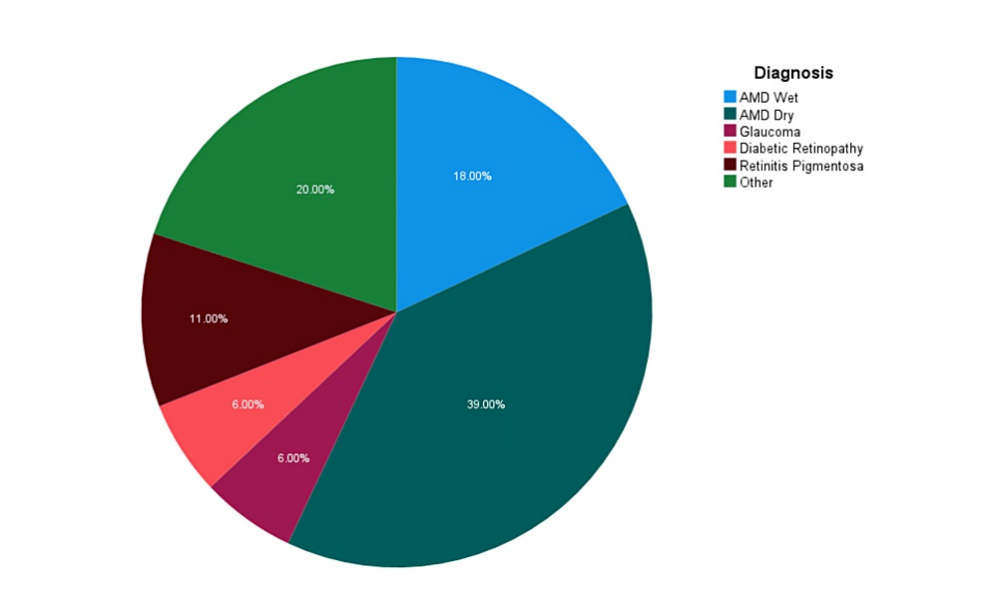
Distribution of causes of LV in the study group AMD, age-related macular degeneration; LV, low vision

A total of 100 LV patients were included in this study, and the daily activities that were affected by their visual impairment are shown in Table 2. Of the participants, 68 were older than 65 years, 50% (n=50) of the participants were male, and 36% (n=36) had attended only mandatory education (Table 3). The median BCVA of the better-seeing eye was 0.96 logMAR (IQR: 0.64-1.3 or moderate-to-severe visual impairment). The majority of patients suffered from AMD; 39% (n=39) had the dry and 18% (n=18) had the wet form of the disease (Figure 1). All patients completed the VFQ-25 questionnaire at baseline, and the mean VFQ-25 score was 49.2 (SD=17.8). At the end of the training protocol, 75 LV patients received an LVA, and 25 did not accept any of the available LVAs. Table 4 shows a comparison between the sociodemographic and clinical characteristics of patients who received at least one LVA versus those who did not. Those suffering from peripheral retinopathies or optic nerve diseases accepted the intervention with LVA significantly better than those suffering from maculopathies (96.3% vs 64.7%, p < 0.001). Except for the type of disease (maculopathy or not), none of the other demographic characteristics was significantly different between the groups. Accordingly, Table 5 shows a comparison between the QoL as evaluated with the VFQ-25 questionnaire and its subscales at the baseline of patients who received at least one LVA versus those who did not. QoL was not found to differ significantly between the groups.

**Table 3:** Patients’ characteristics *Other: retinal detachment-treated (n=3), macular hole (n=2), epiretinal membrane (n=2), Leber congenital amaurosis (n=2), Stargardt disease (n=1), Fuchs’ dystrophy (n=1), congenital X-linked retinoschisis (n=1), Birdshot chorioretinopathy (n=1) AMD, age-related macular degeneration; BCVA, best-corrected visual acuity; LVA, low-vision aid; SD, standard deviation; IQR, interquartile range; VFQ-25, Visual Function Questionnaire-25

Characteristic	N (%)
Age (years)	
<45	16 (16.0)
45-65	16 (16.0)
>65	68 (68.0)
Gender	
Male	50 (50.0)
Female	50 (50.0)
Education	
Mandatory	36 (36.0)
High school	29 (29.0)
University	35 (35.0)
Diagnosis	
AMD	57 (57.0)
Wet form	18 (18.0)
Dry form	39 (39.0)
Retinitis pigmentosa	11 (11.0)
Optic atrophy/neuropathies	7 (7.0)
Glaucoma	6 (6.0)
Diabetic retinopathy	6 (6.0)
Other*	13 (13.0)
VFQ-25 score 16a, mean (SD)	49.2 (17.8)
BCVA of better-seeing eye in logMAR, median (IQR)	0.96 (1.3-0.64)
LVA acceptance	
Yes	75 (75.0)
No	25 (25.0)

**Table 4:** Demographic characteristics of patients who received against those who did not receive LVAs at baseline *P-values < 0.05 indicate a statistically significant difference. **Other: retinal detachment-treated (n=3), macular hole (n=2), epiretinal membrane (n=2), Leber congenital amaurosis (n=2), Stargardt disease (n=1), Fuchs’ dystrophy (n=1), congenital X-linked retinoschisis (n=1), Birdshot chorioretinopathy (n=1). AMD, age-related macular degeneration; BCVA, best-corrected visual acuity; LVA, low-vision aid; IQR, interquartile range

Characteristics	LVA received		P-value*
	No, N=25 (%)	Yes, N=75 (%)	
Age (years)			
<45	1 (4.0)	15 (20.0)	0.17
45-65	4 (16.0)	12 (16.0)	
>65	20 (80.0)	48 (64.0)	
Gender			
Male	12 (48.0)	38 (50.7)	0.82
Female	13 (52.0)	37 (49.3)	
Education			
Mandatory	12 (48.0)	24 (32.0)	0.29
High school	7 (28.0)	22 (29.3)	
University	6 (24.0)	29 (38.7)	
BCVA of better-seeing eye in logMAR, median (IQR)	0.98 (1.3, 0.58)	0.86 (1.3, 0.64)	0.658
Diagnosis			
AMD wet form	5 (20.0)	13 (17.3)	0.07
AMD dry form	18 (72.0)	21 (28.0)	
Optic atrophy/neuropathies	0 (0.0)	7 (9.3)	
Glaucoma	0 (0.0)	6 (8.0)	
Diabetic retinopathy	1 (4.0)	5 (6.7)	
Retinitis pigmentosa	0 (0.0)	11 (14.7)	
Other**	1 (4.0)	12 (16)	
Type of disease			
Maculopathy	24 (96)	44 (62.9)	0.001
Peripheral retinopathy/optic nerve disease	1 (4)	26 (37.1)	

**Table 5:** Comparison between total and subscale scores of the VFQ-25 questionnaire of patients who received an LVA versus those who did not at baseline *P-values < 0 .05 indicate a statistically significant differences AMD, age-related macular degeneration; BCVA, best-corrected visual acuity; LVA, low-vision aid; IQR, interquartile range; SD, standard deviation; VFQ-25, Visual Function Questionnaire-25

	LVA acceptance		P-value*
	No (N=25)	Yes (N=75)	
VFQ-25 16a, mean (SD)	44.8 (17.2)	50.7 (18.7)	0.15
General health, median (IQR)	25 (25-50)	50 (25-75)	0.09
General vision, median (IQR)	40 (40-40)	40 (40-60)	0.07
Ocular pain, median (IQR)	75 (43.8-100)	87.5 (62.5-100)	0.29
Near activities, median (IQR)	25 (10.4-41.7)	41.6 (16.7-50)	0.21
Distance activities, median (IQR)	25 (8.3-50)	33.3 (16.7-50)	0.08
Social functioning, median (IQR)	50 (31.3-75)	50 (37.5-75)	0.57
Mental health, median (IQR)	50 (21.9-56.3)	50 (31.3-68.8)	0.19
Role difficulties, median (IQR)	25 (0-37.5)	25 (12.5-50)	0.17
Dependency, median (IQR)	41.7 (12.5-79.2)	58.3 (25-83.3)	0.41
Driving, median (IQR)	0 (0-0)	0 (0-0)	0.36
Color vision, median (IQR)	50 (25-100)	75 (25-100)	0.59
Peripheral vision, median (IQR)	75 (25-75)	50 (25-100)	0.89

Among patients who did not receive any LVA at baseline, low affordability (40%) and social stigma (28%) were stated as the primary causes of non-acceptance of LVAs. Moreover, some of these patients were above 80 years (50%) old and stated that they can accomplish daily activities such as self-care with the help of their caregivers. Some of them also found such devices difficult to handle, time-consuming, and unnecessary despite the improvement they experienced when using them. A part of these participants stated that they would prefer a more practical LVA for distance vision.

**Figure 2:**
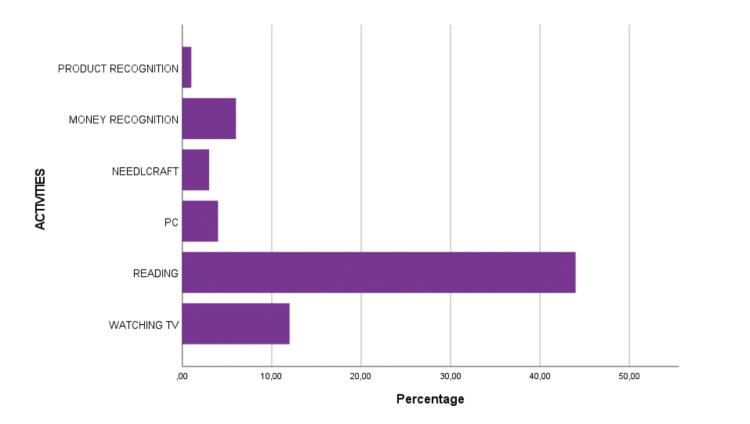
Self-reported activities in which the LVAs were used at one year after the prescription LVA, low-vision aid

Regarding the 75 patients who received an LVA, 76% received an optical visual aid and 98.7% used the LVA they received. Only one participant reported having abandoned the use of the LVA one year after the final session due to worsening visual acuity. Of the patients who used the aid, 62.1% stated that they used the aid often to very often, 77% stated that their QoL was affected slightly positive to positive, and 75.7% stated that they felt to benefit from using the aid. Also, 97.3% of patients would also recommend the aid to other people with the same visual problem (Table 6). The majority of the 11 (14.9%) participants, who stated to rarely use the LVA, reported that the device was not assistive to other daily tasks except for reading. The type of LVAs that were used by participants at one year from baseline and the daily activities in which the LVAs were used are presented in Table 7 and Figure 2, respectively.

**Table 6:** Self-reported use of the prescribed LVA at one year after the prescription *For the 74 patients that used the aid at one year from baseline LV, low vision; LVA, low-vision aid

	N (%)
Type of LVA(s)	
Optical	57 (76.0)
Electronic	14 (18.7)
Optical and electronic	4 (5.3)
Use of the LVA(s) at one year from baseline	
Yes	74 (98.7)
No	1 (1.3)
How often was the LVA(s) used*	
Very often	28 (37.8)
Often	18 (24.3)
Sometimes	17 (23.0)
Rarely	11 (14.9)
Never	0 (0.0)
How was the quality of life affected*	
Positively	31 (41.9)
Slightly positively	26 (35.1)
No difference	17 (23)
Slightly negatively	0 (0.0)
Negatively	0 (0.0)
Do you feel that you benefit from the use of the LVA(s)*	
Yes	56 (75.7)
No	18 (24.3)
Would you recommend the LVA(s) to other people with the same problem*	
Yes	72 (97.3)
No	2 (2.7)

**Table 7:** LVAs that patients (n=74) reported to use at one year after the prescription LVA, low-vision aid

LVAs	N (%)
Handheld lighted magnifier	33 (44.6)
High-plus reading lenses	13 (17.3)
Head-mounted distance glasses, x2.1 magnification for TV	10 (13.3)
Electronic optical reader	9 (12)
Laptop electronic magnifiers	5 (6.7)
Electronic optical reader and head-mounted distance glasses, x2.1 magnification for TV	4 (5.3)

## Discussion

Several studies have found that the decision-making process concerning the use of an assistive device is likely multi-factorial [27-29]. In our study, the selection of an LVA(s) at baseline was not significantly associated with age, gender, visual acuity, education, or underlying cause of LV. Only the type of the disease was found to significantly differ between the groups; specifically, patients suffering from periphery retinopathies or optic nerve diseases accepted better the intervention with an LVA at baseline. Moreover, the compliance with the LVA(s) was not significantly associated with the score of the VFQ-25 questionnaire and its subscales. According to previous studies, age, cause of visual impairment, and visual acuity demonstrated contradictory influences on optical LVAs’ acceptance and usage. Furthermore, the low rate of awareness and motivation along with the worsening of visual function have been identified as possible predictors of LVAs non-use [8]. In a recent scoping review, Lorenzini and Wittich found that studies report high variability rates of people possessing devices but not using them (range: 2.3-50%; mean: 25; SD: 14) [8].

Personal characteristics such as gender, age, and education have been extensively studied by several authors to investigate their potential influence on the use of magnifying LVAs [8]. In a study by Becker et al., it was found that females were more likely to use assistive devices one year from the provision [30]. On the contrary, in another study, gender was not significantly correlated to the perceived benefit from a magnifying LVA [31]. Moreover, any significant correlation between age and LVA use was not revealed [30,31] and age was not found to be an indicator of successful device use. Zammitt et al. reported that elderly patients were more likely to have general associated health problems and limited dexterity skills, which negatively affect the use of LVAs [32]. Furthermore, according to Becker et al., individuals with higher educational levels were more likely to accept LVAs [30]. However, other studies affirmed that the educational level was not a contributing factor to device abandonment [26,33]. In this study, all these factors were not found to affect the uptake of an LVA at baseline.

The effects of diagnosis and visual acuity on the use of LVAs have been studied by several authors, giving divergent conclusions. Though decreasing visual acuity was reported to negatively correlate with LV device compliance [8], it could not be used as a reliable predictor of patient satisfaction or of eventual benefit and was not statistically related to continued use [25]. In our study, we did not detect a statistically significant difference in the acceptance of an LVA across the diagnostic groups. McIlwaine et al. concluded that patients with non-macular disease tended to have lower compliance rates than patients with macular disease; though, they highlighted that etiology could not be used as a reliable predictor of patient satisfaction or of eventual benefit [34]. This is in line with the recent study of Sivakumar et al., who reported that the non-acceptance rate was higher among patients with retinitis pigmentosa [35]. In another study, it was reported that the diagnosis did not affect the continued use of devices in patients with diabetic retinopathy, glaucoma, optic atrophy, myopia, retinitis pigmentosa, and macular degeneration [36]. Moreover, healthier psychological status and higher motivation at the time of the rehabilitation process have been associated with better outcomes [37]. In the current study, compliance with the LVA(s) was not significantly associated with the score of the VFQ-25 questionnaire (Table 5). Though not statistically significant, one of the lowest scores in those who received an LVA was observed in the near activities subscale [score 41.6 (16.7, 50)], which is indicative of the need for assistive devices capturing this field of daily activities.

LVAs aim at improving visual ability and consequently enhance the functional status of the affected individuals. The results of our study are in line with previous studies suggesting that one of the most common forms of intervention is the prescription of magnification devices [26,35,38,39]. Specifically, in our study, the majority of patients who received an LVA preferred a handheld, illuminated magnifier to magnify objects and to facilitate near reading of relatively small prints [26,38,39]. Reading difficulties are frequently reported as one of the most difficult vision-related activities, and reading impairment may worsen during the disease [4,40]. The majority (56%) of the participants in the current study reported reading as the most significant daily activity that was affected by their visual impairment. Reading may be impaired by loss of visual acuity, visual field loss, or degradation of contrast sensitivity. In a prospective study of 63 patients with glaucoma compared with 59 glaucoma suspect controls, patients with glaucoma reported significantly greater reading difficulty compared with controls in nine of the 10 activities. Reading impairment may diminish QoL, especially in elderly patients [41]. Loss of reading ability has been associated with impaired emotional health, mobility, and participation in various activities [41,42].

In our study, 98.7 % of those who received an LVA stated that they used the prescribed device and 62.1% reported using the device often to very often one year after the baseline. A high acceptance rate of the prescribed LV devices was also reported by individuals with LV in the U.S. Department of Veterans Affairs where 84.5% of devices prescribed were still used [43]. On the contrary, only 20% of patients participating in a hospital-based LV program used their LV aid frequently [44]. The working distance that characterizes the use of LVA was found to correlate with the rate of acceptance of the device. In a recent prospective review, the most prescribed LVAs were for near tasks, which is in line with our results. In our study, only 19.9% (n=15) of the participants preferred a distance LVA. Several studies indicated that distance LVAs were characterized by a very high rate of rejection compared to near LVAs [8]. In our study, the distance LVAs were used only for watching TV, as reported by the participants. In another study, 89% of patients were issued with LVAs to assist near vision, only 4% of patients were issued with distance LVAs, and 7% were issued with both [34]. According to Starke et al., optical LVAs tend to be task-specific and easy to use. In our study, participants preferred handheld illuminated magnifiers for near-distance activities [18]. Stronger optical magnifiers typically provide a smaller field of view and are more affected by hand movement than weaker devices. In a recent study by Sivakumar et al., electronic LVAs were found to have a non-acceptance rate of 89% [35]. Low affordability to obtain high-cost electronic devices such as video magnifiers or CCTV was found to be one of the reasons for the non-acceptance of such devices in the same study [18,35].

In this study, 25 participants did not prefer any of the available LVAs. Social stigma, low affordability, and the disconnect between patients’ needs and the availability of appropriately designed LVAs, especially for distance vision, were some of the reported reasons. LVAs for distance activities tend to be abandoned more commonly [45,46]. The limited uptake of LVAs has been previously attributed to the disconnect between user needs and device design characteristics [45]. LVAs are heavy, complicated, and time-consuming, take up too much space, have insufficient magnifying power, and have poor ergonomics, and these consist some of the barriers to the uptake and use of LVAs [8,46]. According to Chaves et al., the acceptance rate is high when assistive devices are well integrated into user’s life as a physical extension of themselves [46].

Appropriate training protocols before the prescription and use of LVAs have proven to be effective in learning how to use LVAs [18,20]. However, an optimal training program in this field is not yet available. The high use of the prescribed LV assistive devices one year after the last session could be attributed to the training of the participants before the use of the device and the patient-centered approach during the session including the record of self-reported significant daily activities that were affected by the LV and the completion of a patient-reported outcome measures such as VFQ-25. Another reason could be the severe visual impairment that most of the participants suffered from (mean BCVA: 0.96 logMAR). To our knowledge, this is the first study examining the acceptance of LVAs in a Greek population with LV investigating also possible factors affecting the decision-making process to choose such an assistive device at baseline. There is a lack of awareness among eye care practitioners about LV rehabilitation [9,47-50], and studies investigating the patients’ perspectives using individualized approaches are few. Thus, it is important to investigate such parameters so that clinicians can develop strategies to improve compliance with the use of the LVAs.

Limitations of the study are the non-availability of all types of LVAs that could meet some of the patients’ needs. However, a wide range of LVAs was provided, and the majority of participants benefitted from the use of these devices. Furthermore, a longitudinal follow-up extended for more than one year after the final session could provide a valuable feedback on the use of the LVAs. Most of the ocular diseases causing LV exhibit a progressive course, leading to changes in visual requirements and demanding a reassessment and re-prescription of the suitable LVA(s) [50]. The findings of the current study on the effect of the examined variables such as age and visual acuity on the decision-making process for the uptake of LVAs are indicative, but a larger, controlled study including different groups of patients based on these parameters might provide more information. Finally, the current study did not cover other aspects of LV rehabilitation apart from LVAs.

## Conclusions

The majority of LV patients continued to use and benefit from the prescribed LVA one year after the training. Among several demographic factors, only the type of the disease was found to influence the acceptance of LVAs at baseline. A more individualized approach based on LV patients’ needs along with the provision of appropriate training before the prescription of the LVAs is of high significance to achieve high rates of compliance with the use of LVAs.

## Appendices

Compliance with the use of LVA(s) after a training protocol

Script:

Thank you for agreeing to take part in this study. This survey will ask you about your views on the LVA(s) you received.

All responses that you provide will remain confidential and no identifying information will be published. This survey is expected to take approximately 5 minutes.

Participant ID:                         ____________________

Date:                                        ____________________

1. Which type of LVA(s) did you received?

Optical

Electronic

Optical and Electronic

2. Have you used the LVA(s)?

Yes

No

3. How often have you used the LVA(s)?

Very often

Often

Sometimes

Rarely

Never

4. How was the quality of your life affected by the use of the LVA(s) [25]?

Positively

Slightly positively

No difference

Slightly negatively

Negatively

5. Do you feel that you benefit from the use of the LVA(s) [25]?

Yes

No

6. Would you recommend the LVA(s) to other people with the same problem [25]?

Yes

No

## References

[1] Cherinet FM, Tekalign SY, Anbesse DH, Bizuneh ZY (2018). Prevalence and associated factors of low vision and blindness among patients attending St. Paul's Hospital Millennium Medical College, Addis Ababa, Ethiopia. BMC Ophthalmol.

[2] Bourne RR, Stevens GA, White R (2013). Causes of vision loss worldwide, 1990-2010: a systematic analysis. Lancet Glob Health.

[3] World Health Organisation (2023). Management of low vision in children : report of a WHO consultation, Bangkok, 23-24 July 1992. https://apps.who.int/iris/handle/10665/61105.

[4] Shah P, Schwartz SG, Gartner S, Scott IU, Flynn HW Jr (2018). Low vision services: a practical guide for the clinician. Ther Adv Ophthalmol.

[5] Woolhead G, Calnan M, Dieppe P, Tadd W (2004). Dignity in older age: what do older people in the United Kingdom think?. Age Ageing.

[6] Scott IU, Smiddy WE, Schiffman J (1999). Quality of life of low-vision patients and the impact of low-vision services. Am J Ophthalmol.

[7] de Boer MR, Langelaan M, Jansonius NM, van Rens GH (2005). [Referral for rehabilitation in case of permanent visual handicap; guideline of the Dutch Society of Ophthalmology]. Ned Tijdschr Geneeskd.

[8] Trauzettel-Klosinski S (2010). Rehabilitation for visual disorders. J Neuroophthalmol.

[9] Lorenzini MC, Wittich W (2020). Factors related to the use of magnifying low vision aids: a scoping review. Disabil Rehabil.

[10] Casten RJ, Maloney EK, Rovner BW (2005). Knowledge and use of low vision services among persons with age-related macular degeneration. J Vis Impair Blind.

[11] Lighthouse International (1995). The Lighthouse National Survey on Vision Loss: The Experience, Attitudes and Knowledge of Middle-aged and Older Americans. New York: Author.

[12] Gothwal VK, Sharma S (2023). What are the reasons for abandonment of low vision devices prescribed in a large tertiary eye care centre?. Ophthalmic Physiol Opt.

[13] Binns AM, Bunce C, Dickinson C (2012). How effective is low vision service provision? A systematic review. Surv Ophthalmol.

[14] Binns A, Bunce C, Dickinson C (2023). Low vision service outcomes: a systematic review. https://media.rnib.org.uk/documents/LOVESME_lit_reviewP.pdf.

[15] Dickinson C, Linck P, Tudor-Edwards R (2011). A profile of low vision services in England: the Low Vision Service Model Evaluation (LOVSME) project. Eye (Lond).

[16] Markowitz SN (2006). Principles of modern low vision rehabilitation. Can J Ophthalmol.

[17] Markowitz SN (2016). State-of-the-art: low vision rehabilitation. Can J Ophthalmol.

[18] Starke SD, Golubova E, Crossland MD, Wolffsohn JS (2020). Everyday visual demands of people with low vision: a mixed methods real-life recording study. J Vis.

[19] Burggraaff MC, van Nispen RM, Melis-Dankers BJ, van Rens GH (2010). Effects of standard training in the use of closed-circuit televisions in visually impaired adults: design of a training protocol and a randomized controlled trial. BMC Health Serv Res.

[20] Stelmack JA, Tang XC, Reda DJ, Rinne S, Mancil RM, Massof RW (2008). Outcomes of the Veterans Affairs Low Vision Intervention Trial (LOVIT). Arch Ophthalmol.

[21] Khoshnood B, Mesbah M, Jeanbat V, Lafuma A, Berdeaux G (2010). Transforming scales of measurement of visual acuity at the group level. Ophthalmic Physiol Opt.

[22] Labiris G, Katsanos A, Fanariotis M, Tsirouki T, Pefkianaki M, Chatzoulis D, Tsironi E (2008). Psychometric properties of the Greek version of the NEI-VFQ 25. BMC Ophthalmol.

[23] (2007). Laboratory of Experimental Ophthalmology of Aristotle University, Thessaloniki, Greece. National Eye Institute Visual Functioning Questionnaire-25 (VFQ-25). https://www.rand.org/content/dam/rand/www/external/health/surveys_tools/vfq/vfq25survey_greek.pdf.

[24] Kelleher DK (1976). Training low vision patients. J Am Optom Assoc.

[25] Demirkilinc E, Palamar M, Uretmen O (2013). Low vision aids: the effectiveness of low vision rehabilitation. Turk Klin Tip Etigi Hukuku Tarihi.

[26] Dougherty BE, Kehler KB, Jamara R, Patterson N, Valenti D, Vera-Diaz FA (2011). Abandonment of low-vision devices in an outpatient population. Optom Vis Sci.

[27] Tomita MR, Mann WC, Fraas LF (2004). Predictors of the use of assistive devices that address physical impairments among community-based frail elders. J Appl Gerontol.

[28] Kraskowsky LH, Finlayson M (2001). Factors affecting older adults' use of adaptive equipment: review of the literature. Am J Occup Ther.

[29] Scherer MJ, Sax C, Vanbiervliet A, Cushman LA, Scherer JV (2005). Predictors of assistive technology use: the importance of personal and psychosocial factors. Disabil Rehabil.

[30] Becker S, Wahl HW, Schilling O, Burmedi D (2005). Assistive device use in visually impaired older adults: role of control beliefs. Gerontologist.

[31] Leat SJ, Fryer A, Rumney NJ (1994). Outcome of low vision aid provision: the effectiveness of a low vision clinic. Optom Vis Sci.

[32] Zammitt N, O’Hare A, Mason J (1999). Use of low vision aids by children attending a centralized multidisciplinary visual impairment service. J Vis Impair Blind.

[33] Goldstein RB, Dugan E, Trachtenberg F, Peli E (2007). The impact of a video intervention on the use of low vision assistive devices. Optom Vis Sci.

[34] McIlwaine GG, Bell JA, Dutton GN (1991). Low vision aids--is our service cost effective?. Eye (Lond).

[35] Sivakumar P, Vedachalam R, Kannusamy V (2020). Barriers in utilisation of low vision assistive products. Eye (Lond).

[36] Nilsson UL, Nilsson SE (1986). Rehabilitation of the visually handicapped with advanced macular degeneration. A follow-up study at the Low Vision Clinic, Department of Ophthalmology, University of Linköping. Doc Ophthalmol.

[37] Mitchell J, Bradley C (2006). Quality of life in age-related macular degeneration: a review of the literature. Health Qual Life Outcomes.

[38] Hooper P, Jutai JW, Strong G, Russell-Minda E (2008). Age-related macular degeneration and low-vision rehabilitation: a systematic review. Can J Ophthalmol.

[39] Robillard N, Overbury O (2006). Quebec model for low vision rehabilitation. Can J Ophthalmol.

[40] Rinnert T, Lindner H, Behrens-Baumann W (1999). [At home utilization of low-vision aids by the visually impaired]. Klin Monbl Augenheilkd.

[41] Nguyen AM, van Landingham SW, Massof RW, Rubin GS, Ramulu PY (2014). Reading ability and reading engagement in older adults with glaucoma. Invest Ophthalmol Vis Sci.

[42] Hassell JB, Lamoureux EL, Keeffe JE (2006). Impact of age related macular degeneration on quality of life. Br J Ophthalmol.

[43] Watson GR, De l'Aune W, Stelmack J, Maino J, Long S (1997). National survey of the impact of low vision device use among veterans. Optom Vis Sci.

[44] Elliott AJ (1989). Poor vision and the elderly--a domiciliary study. Eye (Lond).

[45] Golubova E, Starke SD, Crossland MD, Wolffsohn JS (2021). Design considerations for the ideal low vision aid: insights from de-brief interviews following a real-world recording study. Ophthalmic Physiol Opt.

[46] Gobeille MR, Malkin AG, Jamara R, Ross NC (2018). Utilization and abandonment of low vision devices prescribed on a mobile clinic. Optom Vis Sci.

[47] Chaves ES, Cooper RA, Collins DM, Karmarkar A, Cooper R (2007). Review of the use of physical restraints and lap belts with wheelchair users. Assist Technol.

[48] Colenbrander A (2018). Vision rehabilitation is part of AMD care. Vision (Basel).

[49] Keeffe JE, Lovie-Kitchin JE, Taylor HR (1996). Referral to low vision services by ophthalmologists. Aust N Z J Ophthalmol.

[50] Das K, Gopalakrishnan S, Dalan D, Velu S, Ratra V, Ratra D (2019). Factors influencing the choice of low-vision devices for visual rehabilitation in Stargardt disease. Clin Exp Optom.

